# Machine learning-assisted chemometric analysis of salivary ATR-FTIR spectra for the diagnosis of oral submucous fibrosis

**DOI:** 10.3389/fonc.2026.1927981

**Published:** 2026-10-19

**Authors:** Priya Shree, Yogender Aggarwal, Manish Kumar, Priyank Saxena, Narendra Nath Singh, Akhilesh Chandra, Prerna Srivastava, Somili Nag, Arpita Rai

**Affiliations:** 1 Vananchal Dental College and Hospital, Garhwa, India; 2 Birla Institute of Technology, Ranchi, India; 3 Rajendra Institute of Medical Sciences, Ranchi, India; 4 Banaras Hindu University, Varanasi, India

**Keywords:** decision tree, Fourier transform infrared spectroscopy, machine learning, oral submucous fibrosis, random forest, support vector machine, XGBoost

## Abstract

**Background:**

Oral submucous fibrosis (OSMF) is a chronic, potentially malignant disorder strongly linked to areca nut chewing. Clinical diagnosis is challenging, and histopathology, though the gold standard, is invasive and subject to inter-observer variability. Saliva analysis using Fourier Transform Infrared (FTIR) spectroscopy offers a promising non-invasive alternative. This study evaluated salivary ATR-FTIR spectral analysis integrated with machine learning for classification of OSMF, individuals with adverse oral habits, and healthy controls.

**Methodology:**

Unstimulated saliva samples were collected from 450 participants comprising 150 patients with clinically diagnosed OSMF, 150 individuals with adverse oral habits without clinical evidence of OSMF, and 150 healthy controls. After centrifugation, the saliva supernatant was analyzed by ATR-FTIR spectroscopy over 400–4000 cm^−^¹. Spectra underwent baseline correction and normalization followed by Savitzky–Golay smoothing. For machine-learning analysis, Standard Scaler and PCA were incorporated within the classification pipeline. Decision Tree, Random Forest, support vector machine (SVM), and XGBoost classifiers were evaluated using stratified 10-fold cross-validation. Hyperparameter optimization and performance estimation used the same cross-validation framework. An independent cohort of 90 participants was used for validation.

**Results:**

XGBoost showed the highest cross-validation performance, with accuracies of 90.62% for Normal versus Habit, 97.67% for Normal versus OSMF, and 91.30% for three-class classification. Statistical analysis identified 157 nominally significant wavenumbers (p<0.05) within a continuous region from 1581 to 2142 cm^−^¹ corresponding to protein and collagen bands. Principal component analysis showed that PC1 and PC2 explained 89.26% and 6.95% of the total variance, respectively (96.21% cumulatively). In the independent validation cohort, accuracy was 80.0% for OSMF versus Normal and 71.7% for Habit versus Normal.

**Conclusion:**

Salivary ATR-FTIR combined with machine learning demonstrated promising classification performance and preliminary performance in an independent cohort. The identified spectral region is biologically plausible in the context of altered salivary biochemical composition associated with inflammation and fibrosis, but it should not be regarded as a validated disease-specific biomarker. Salivary ATR-FTIR is best considered a complementary, non-invasive screening and risk-stratification approach rather than a replacement for clinical/oral examination or histopathological assessment when biopsy is indicated. Larger, diverse, multicenter studies with rigorous validation are needed before clinical implementation.

## 1 Introduction

Oral submucous fibrosis (OSMF) is a potentially malignant disorder commonly observed in the Indian subcontinent. It has a multifactorial etiology, with habitual chewing of areca nut (betel nut) being the most prominent contributing factor. Schwartz first described this condition in 1952 (1). Globally, millions of people consume areca nut, placing them at risk for developing OSMF. The reported prevalence of OSMF ranges from 0.03% to 30%, regardless of areca nut usage (2). Clinically, OSMF presents a significant challenge due to its resemblance to other mucocutaneous conditions, which often leads to misdiagnosis and inappropriate treatment, further complicating patient outcomes (3). The Oral Medicine Society recommends a comprehensive diagnostic approach that includes a detailed history of areca nut use, characteristic clinical features, histopathological examination, and auxiliary diagnostic methods such as autofluorescence imaging, toluidine blue staining, and exfoliative cytology. Despite these options, histopathological examination remains the gold standard for diagnosis (1). However, it is invasive, subjective, and prone to considerable inter-observer variability, resulting in inconsistent grading and reduced reproducibility. Moreover, the limited mouth opening characteristic of OSMF often hinders the ability to perform proper biopsies and histological evaluations (4, 5). These limitations highlight the urgent need for more efficient, objective, and non-invasive diagnostic tools. Saliva is a complex biological fluid, consisting of approximately 98% water along with various enzymes, electrolytes, minerals, proteins, nucleic acids, antibodies, microorganisms, and cellular components. Due to its rich composition, saliva can reflect both local and systemic health conditions, making it a valuable medium for disease diagnostics, especially for disorders affecting the oral cavity. Saliva also offers practical advantages: it is easily accessible, enables non-invasive and repeatable sampling, and does not require complex or expensive equipment for collection (6). Furthermore, its biochemical constituents show significant potential in patient stratification and disease monitoring. Fourier Transform Infrared (FTIR) spectroscopy has emerged as a promising diagnostic modality owing to its non-invasive nature, cost-effectiveness, rapid analysis time, and safety. FTIR-based techniques enable the detection of subtle biochemical changes in biological samples, such as saliva, potentially offering an alternative to traditional diagnostic methods. However, despite its promise, the broader applicability and long-term diagnostic efficacy of FTIR remain underexplored, necessitating further research and clinical validation (7, 8).

In this study, we utilized FTIR spectroscopy to analyze saliva samples from three groups: healthy individuals (control), individuals with adverse oral habits (Habit), and patients diagnosed with OSMF. FTIR spectral profiles were obtained across a range of 400–4000 cm^−^¹.The key differences in the spectral data were analyzed using various machine learning models, including, Support Vector Machine (SVM), XGBoost, and Random Forest, to classify the samples separately for diagnosis as well as screening for the disease. The performance of these models was evaluated in terms of accuracy and reliability. The initial findings demonstrate that these models can distinguish between control and diseased samples and also between Control and Habit effectively. With further validation using blinded or unknown samples, these models hold the potential to serve as efficient, non-invasive tools for screening and accurate diagnosis of OSMF.

## 2 Materials and methods

### 2.1 Ethical statement

This study was conducted with the authorization of the Institutional Ethics Committee IEC Reg. No- ECR/769/INST/JH/2015/RR-18.

### 2.2 Study population

The research was a collaborative effort between the Department of Oral Medicine and Radiology at the Dental Institute, RIMS Ranchi, which oversaw participant recruitment, and the Birla Institute of Technology, Ranchi, where FTIR testing was meticulously carried out. A total of 450 participants were enrolled and recruited equally into three groups: Group A consisted of patients with clinically confirmed OSMF, Group B included participants with adverse oral habits but without any clinical evidence of Oral submucous fibrosis, and Group C consisted of healthy individuals serving as controls. All clinical examinations were performed by a single trained clinician to maintain consistency in diagnosis and grading of Oral submucous fibrosis. Prior to the commencement of the study, the clinician underwent training and standardization for the examination protocol to ensure uniform application of the diagnostic criteria and grading system. All participants provided written informed consent to join the study. Additionally, comprehensive demographic information, along with detailed histories of tobacco and alcohol use, including duration, frequency, and quantity, was collected from each participant.

### 2.3 Participant selection

The diagnosis of Oral submucous fibrosis in this study was established through detailed clinical examination using the classification described by Kerr et al. (9) Patients were categorized into different grades based on characteristic clinical features including mucosal blanching, burning sensation, fibrotic bands, leathery mucosa, depapillation of the tongue, and measurement of inter-incisal mouth opening. According to this classification, Grade 1 represents mild disease with mouth opening ≥35 mm, Grade 2 corresponds to moderate disease with mouth opening between 20–35 mm, and Grade 3 indicates severe disease with mouth opening <20 mm. Higher grades include cases associated with other potentially malignant disorders or malignant transformation. Biopsy and histopathological examination were performed in cases where clinical findings suggested the possibility of epithelial dysplasia or malignant transformation. Participants in whom histopathological examination revealed epithelial dysplasia or oral squamous cell carcinoma were excluded from the study to ensure that the analysis included only clinically diagnosed OSMF cases without malignant transformation.

Participants diagnosed with Oral submucous fibrosis were included in Group A if they reported current or previous use of smokeless tobacco and/or areca nut, complained of burning sensation of the mouth or difficulty in chewing and swallowing, and showed clinical features such as restricted mouth opening, mucosal blanching, stiffness, or the presence of vertical fibrous bands. In group A, patients with significant systemic medical conditions, those already undergoing treatment for OSMF, or those presenting with concomitant dental conditions such as pericoronitis or other causes of trismus were excluded. Participants with other conditions that could lead to restricted mouth opening, including temporomandibular joint disorders, oral infections, or trauma, were also excluded. In group B, participants with adverse oral habits, such as areca nut and/or tobacco use, but without clinical evidence of OSMF were included. These patients were matched for age (within ± 2 years) and duration of tobacco habits with the OSMF patients. Patients with previous history of any oral potentially malignant or malignant lesions were excluded. In group 3, healthy individuals without clinical evidence of OSMF or other oral pathology or any current or previous habit of tobacco (any form)/areca nut were included. These patients were also age matched (within ± 2 years) to Group 1 patients.

### 2.4 Sample size calculation

The minimum sample size for the study was calculated using PASS software (Utah, USA). Based on an estimated prevalence of 5% (0.3%–6.42%) for Oral submucous fibrosis, the minimum required sample size was determined to be 240 participants. The sensitivity and specificity for the null hypothesis were set at 50% and 90%, respectively, with a significance level of p < 0.05. Following the rule of thumb and to improve the robustness of the machine learning models, the total sample size was increased to 450 participants, which were used during the training phase and divided into three groups: OSMF patients, individuals with adverse oral habits, and healthy controls. Additionally, 90 independent samples (30 in each group) were included for the testing phase to evaluate the performance of the developed models.

### 2.5 Sample collection

A consistent protocol was used to collect unstimulated whole saliva samples. Participants were asked to rinse their mouths with water and avoid eating, drinking, smoking, using smokeless tobacco, or using oral hygiene products for at least one hour before sample collection. Samples were gathered in the morning between 9 am and 10 am, with patients seated comfortably and spitting saliva into a plastic container as it accumulated. Approximately 3–4 ml of saliva was collected from each participant. Samples were centrifuged (2500 × g for 15 minutes at 4 °C), and supernatants were aliquoted (250 µl in 1 ml microcentrifuge tubes) and stored at -80 °C for further analysis. The use of the supernatant was intended to provide a more reproducible soluble sample fraction and to reduce the contribution of particulate material and cellular debris to spectral measurements. The remaining stored sample was retained for repeat evaluation if required in case of any problem during the initial analysis and may also be considered for future research, subject to the original informed consent and applicable ethical requirements.

### 2.6 FTIR spectroscopy method

FTIR spectroscopy was performed on all saliva samples from all three groups. An IR Prestige-21 (Shimadzu Corp, Japan) spectrometer recorded the IR spectra in the 400-4000cm^-1^ range. The spectral range of 400–4000 cm^−^¹ was selected to capture a broad range of vibrational features arising from the major biochemical constituents present in saliva, including proteins, lipids, carbohydrates, and other organic components. In ATR mode, MIRacle™ A Single Reflection horizontal ATR accessory from PIKE Technologies which contains a Zinc Selenide Crystal with a refractive index value 2.4was used. The samples were thawed and brought to room temperature. A micropipette was used to place one drop of the thawed saliva supernatant onto the sample table, after which the spectrum was recorded. Air spectra served as the background prior to each sample analysis. Both background and sample spectra were recorded at room temperature (approximately 21-23 °C), with a spectral resolution of 4cm^-1^, and each measurement included 32 scans. All IR spectra, reflecting the infrared absorbance (abs. units) as a function of the wave number, were obtained through at least three consecutive tests with 3–5 repetitions each. The original FTIR spectra were normalized and baseline-corrected using OPUS 7.x software.

### 2.7 Spectral data processing

An air spectrum was recorded under identical instrumental conditions before each sample analysis and was used as the background reference. The background measurement accounted for atmospheric and instrumental contributions, and the recorded background spectrum was subtracted during sample acquisition. For pre-processing, raw spectral data were loaded from Excel files, transposed to yield sample-by-wavenumber matrices, and wavenumber labels assigned as np.linspace(400, 4000, 1001). Savitzky-Golay smoothing (window length=11, polynomial order=3) was applied to each sample to suppress instrument noise while preserving peak morphology.

Further, data cleaning was performed to ensure the quality and integrity of the FTIR spectral dataset. This process involved handling missing or inconsistent values. Thereafter, feature standardization was performed to have a zero mean and unit variance. This step ensured uniform feature scaling, improving the performance and convergence of machine learning classifiers.

Subsequent data processing and visualization were performed by Python 3.8. The spectrum was plotted with wave number (cm^−1^) on the X-axis and transmittance (absorbance)on the Y-axis. Spectral graphs for each group were obtained and compiled.

### 2.8 PCA

In this study, the input to Principal Component Analysis (PCA) consisted of scaled FTIR spectral data, where each row represented a salivary sample and each column corresponded to absorbance values at specific wavenumbers, resulting in a high-dimensional matrix. PCA was applied to reduce this large number of correlated features to a smaller set of uncorrelated principal components that retained 99% of the original data variance. The output of PCA with reduced feature set was subsequently used as input for training various machine learning classifiers for disease screening and diagnosis.

### 2.9 Statistical analysis

One-way ANOVA was performed independently at each of the 1,001 wavenumber positions to identify spectral regions differing significantly between groups (eg normal and habit). The Bonferroni-corrected threshold was set at α/1001 = 4.99×10^−5^.

### 2.10 Machine learning framework

All classifiers were embedded in a standardized pipeline: Standard Scaler → PCA (n=10) → Classifier. Four classifiers were evaluated: Decision Tree (max_depth=10), Random Forest (500 trees, max_depth=10), SVM (RBF kernel, C = 10), and XGBoost (hyperparameters tuned via Grid Search CV over n estimators ∈ {100,200,300}, max depth∈ {3,5,7}, learning rate ∈ {0.05,0.1,0.2}). These four classifiers were selected to compare different machine-learning approaches for classification of the FTIR spectral data. Decision Tree provides a tree-based classification approach, while Random Forest uses an ensemble of multiple decision trees. SVM was included to evaluate classification using the RBF kernel, and XGBoost was used as a boosting-based tree method. This comparison allowed the performance of different classification approaches to be evaluated using the same spectral dataset. The preprocessing-classification pipeline (StandardScaler → PCA (10 components) → Classifier) was passed directly to GridSearchCV with stratified 10-fold cross-validation, so the scaler and PCA were fitted independently on the training portion of each fold and applied to the corresponding validation portion, preventing validation-fold leakage during preprocessing. However, hyperparameter tuning and the reported cross-validation accuracy used the same 10-fold framework rather than a fully nested design, and the subsequent 80/20 test split was generated after tuning, so test samples had already been part of the cross-validation search. The reported cross-validation and test accuracies should therefore be interpreted as upper-bound estimates rather than results of a strictly nested cross-validation procedure.Accuracy, precision, recall, F1-score, and confusion matrices were calculated for the test set. An independent external cohort was additionally used to evaluate model performance on new samples (**Table 1**).

**Table 1:** The specific Python libraries used at each stage of the analysis pipeline were as follows:.

Pipeline stage	Library/function	Purpose
Data handling	NumPy, pandas	Loading, reshaping, and transposing raw spectral Excel files into sample-by-wavenumber matrices.
Preprocessing	SciPy (savgol_filter)	Savitzky–Golay smoothing of raw absorbance spectra.
Scaling & dimensionality reduction	scikit-learn (StandardScaler, PCA)	Feature standardization and principal component analysis (n=10 components).
Classification	scikit-learn (DecisionTreeClassifier, RandomForestClassifier, SVC), XGBoost (XGBClassifier)	Model training for all four classifiers.
Model tuning & validation	scikit-learn (GridSearchCV, StratifiedKFold, cross_val_score)	Hyperparameter tuning and 10-fold cross-validation.
Visualization	Matplotlib, Seaborn	Spectral plots, PCA scatter plots, confusion matrices, and ROC curves.

### 2.11 Evaluation of performance and diagnostic accuracy

To evaluate the performance and diagnostic accuracy of the machine learning models, Receiver Operating Characteristic (ROC) curves were plotted. The Area under the Curve (AUC) was calculated to quantify the discriminatory power of the model in distinguishing OSMF samples from those of healthy control groups and adverse habits from control.

### 2.12 External validation

For external validation of the proposed model, a total of 90 new patients (30 patients clinically diagnosed with Oral submucous fibrosis (OSMF), 30 individuals with oral habits such as tobacco usage and 30 healthy individuals without any oral lesions, acting as normal controls) were enrolled and saliva samples were collected. FTIR spectroscopy was done for these samples as described above. Sensitivity, specificity and overall diagnostic accuracy was calculated.

## 3 Results

### 3.1 Demographic detail

Saliva from a total of 450 patients was collected for the present study. The detailed description of dataset composition is given in **Table 2**.

**Table 2:** Dataset composition.

Variable	OSMF (n=150)	Habit (n=150)	Normal (n=150)
Sample IDs	OSMF1–OSMF150	H1–H150	N1–N150
Age (years), mean ± SD	35.7 ± 10.53	34.1 ± 8.9	33.8 ± 9.6
Male, n (%)	147 (98)	136 (90.6)	140 (93.3)
Female, n (%)	3 (2)	14 (9.4)	10 (6.7)
Tobacco frequency (times/day) Mean ± SD	6.4 ± 2.9	5.8 ± 2.7	–
Tobacco-use duration (years) Mean ± SD	9.1 ± 5.0	8.2 ± 4.6	–
Description	Patients diagnosed with Oral Submucous Fibrosis	Patients with areca nut/tobacco chewing habits	Healthy oral mucosa, no habits or pathology

A total of 450 participants were included in the study, with 150 participants each in the OSMF, Habit, and Normal groups. The mean age was 35.7 ± 10.53 years in the OSMF group, 34.1 ± 8.9 years in the Habit group, and 33.8 ± 9.6 years in the Normal group. The OSMF group included 147 males (98.0%) and 3 females (2.0%), while the Habit group included 136 males (90.6%) and 14 females (9.4%). The Normal group included 140 males (93.3%) and 10 females (6.7%). Statistically, no significant difference was observed between the mean ages of OSMF group, Habit group, and Normal group (p = 0.802745).

Among participants with tobacco use, the mean tobacco frequency was 6.4 ± 2.9 times per day in the OSMF group and 5.8 ± 2.7 times per day in the Habit group. The mean duration of tobacco use was 9.1 ± 5.0 years in the OSMF group and 8.2 ± 4.6 years in the Habit group. Tobacco frequency and duration were not applicable to the Normal group because participants in this group had no tobacco or other adverse habits.

### 3.2 FTIR interpretation

FTIR absorbance spectra were collected from saliva samples across three groups. Each sample was represented by 1,001 absorbance readings spanning wavenumbers 400–4000 cm^−^¹ (linearly spaced, step ~3.6 cm^−^¹). After pre-processing, mean noise removal was 0.05% with maximum peak correction of 0.33% (**Figure 1**).

**Figure 1:**
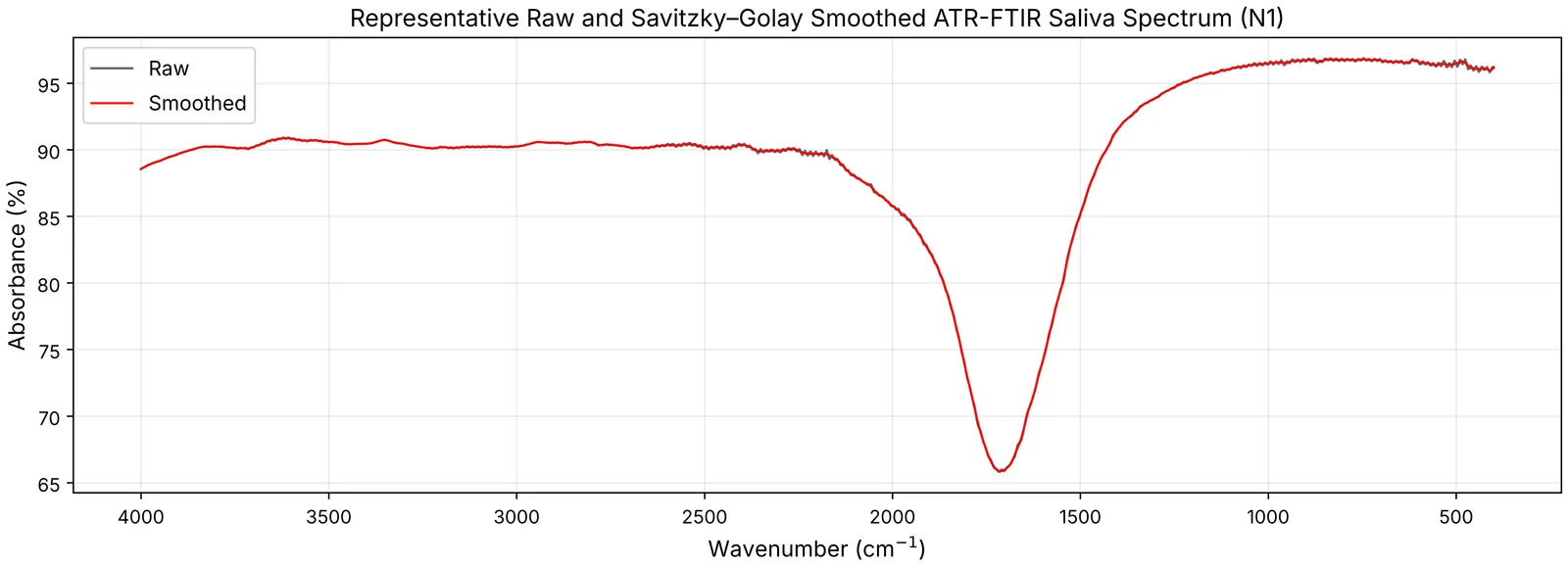
Representative raw and Savitzky–Golay smoothed ATR-FTIR saliva spectrum.

These noise metrics were calculated as the mean and maximum absolute difference between each raw and Savitzky–Golay-smoothed absorbance value across all wavenumbers, quantifying the magnitude of high-frequency instrumental noise removed by smoothing. These noise metrics were computed using Equations 1 and 2.

(1) $$Noise_{mean}\;=\;\left(1/N\right)\sum \left|A_{raw}\left(v_{i}\right)-A_{smooth}\left(v_{i}\right)\right|$$

(2) $$Noise_{mean}\;=\;\max\left|A_{raw}\left(v_{i}\right)-A_{smooth}\left(v_{i}\right)\right|$$

where A_raw_(ν) and A_smooth_(ν) are the raw and Savitzky–Golay-smoothed absorbance values at wavenumber ν, i = 1,…,N, and N = 1001 is the total number of wavenumber points across the 400–4000 cm^−^¹ range.

ANOVA identified 157 out of 1,001 wavenumbers as significant at p<0.05 (15.7%), forming one continuous spectral region: 1581–2142 cm^−^¹ (**Figure 2**). The ten most significant wavenumbers clustered at 1998–2045 cm^−^¹ (F≈9.0, p≈0.003). Although 157 wavenumbers showed nominal significance (p<0.05), none remained significant after Bonferroni correction, suggesting subtle biochemical differences that warrant further investigation. The 1581–1700 cm^−^¹ portion encompasses predominantly protein-associated Amide II and Amide I vibrations, while the 1700–2000 cm^−^¹ region includes carbonyl-related (C=O stretching lipid vibrations) and other molecular vibrations that may arise from lipid and protein constituents. The 2000–2142 cm^−^¹ sub-region does not correspond to a classical Amide band but is more consistent with combination and overtone vibrations arising from underlying protein, lipid, and carbohydrate constituents, and its biological interpretation should therefore be considered tentative pending further spectral assignment work.

**Figure 2:**
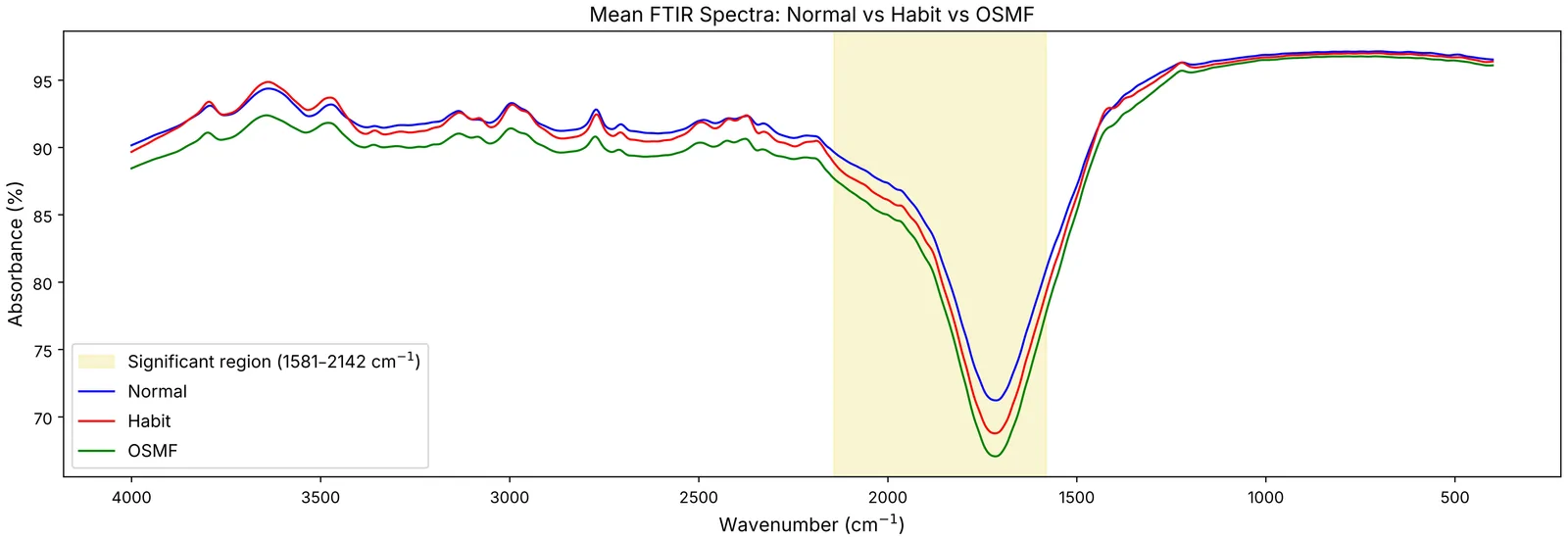
Mean FTIR spectra for Normal (blue), Habit (red), and OSMF (green) groups. Yellow shading marks the statistically significant region (1581–2142 cm^−^¹).

### 3.3 PCA

Systematic evaluation of PCA showed ten components with consistently optimized performance across all comparisons. The PCA scatter plot visualizes the distribution of data points based on the first two principal components (PC1 and PC2). The plot highlights the clustering and overlaps between these groups, with some regions showing mixed colors, indicating overlapping characteristics. This visualization effectively captures the variance and relationships among the groups based on the principal components (**Figure 3**). PC1 and PC2 accounted for 89.26% and 6.95% of the total spectral variance, respectively (96.21% cumulative), indicating that the dominant source of separation among Normal, Habit, and OSMF samples is captured within the first two components, largely reflecting broad differences in overall absorbance intensity and the amide/lipid-associated spectral region.

**Figure 3:**
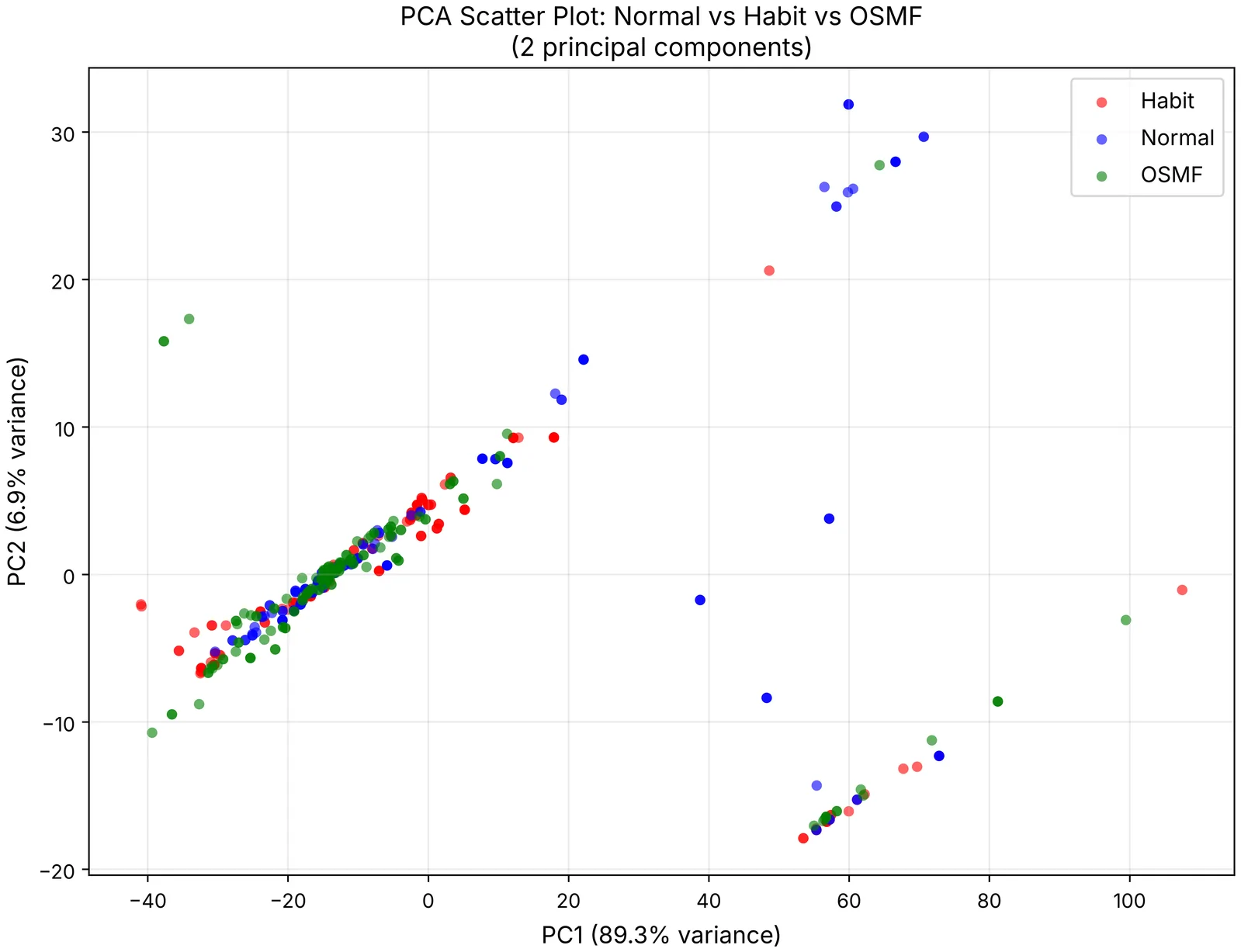
PCA scatter plot showing separation of Normal (blue), Habit (red), and OSMF (green) samples across the first two principal components.

### 3.4 Model performance

A three-class model was developed combining all three patient groups, enabling simultaneous discrimination without prior knowledge of the patient category. Among the evaluated classifiers, XGboost consistently demonstrates the highest performance, achieving 91.3% ± 3.69% training accuracy and 87.78% testing accuracy. The 10-fold cross-validation accuracy of XGboost was 83.5%. These findings indicate promising classification performance in the study dataset, while the external validation results provide preliminary evidence of generalizability. Random forest and decision tree also performed reasonably well with training accuracy of 79.49% ± 6.09% and 75.93% ± 6.55% respectively and 10-fold cross-validation accuracy of 10-79.5% and 75.9% respectively. SVM has the lowest performance across all tasks, with testing accuracy of 57.46% ± 5.89%, the 10-fold cross-validation accuracy of 57.5%; suggesting it’s not suitable for capturing the complex patterns in this dataset. Overall, XG boost was the most effective classifier effectively capturing the important patterns in the data while maintaining good generalization ability (**Table 3**). To assess the contribution of preprocessing, the same pipeline (Standard Scaler → PCA (n=10) → XGBoost, 10-fold CV) was additionally run on the raw, unsmoothed spectra, yielding a mean cross-validation accuracy of 89.07% (± 4.97%), compared with 91.30% (± 4.53%) for Savitzky–Golay-smoothed spectra, confirming that smoothing provided a modest but consistent improvement in classification performance.

**Table 3:** Three-class results (PCA n=10).

Model	CV Accuracy	CV Std	Test Accuracy
Decision Tree	75.93%	± 6.55%	—
Random Forest	79.49%	± 6.09%	—
SVM	57.46%	± 5.89%	—
XGBoost (tuned) ★	91.30%	± 3.69%	87.78%

★ = best model.

The confusion matrix analysis demonstrated superior performance of the XGBoost classifier compared with Decision Tree, Random Forest, and SVM models (**Figure 4**). XGBoost achieved the highest overall accuracy (91.3%), correctly classifying 410 of 449 samples, followed by Random Forest (90.0%) and Decision Tree (86.9%). In contrast, SVM exhibited substantially lower classification accuracy (63.5%). XGBoost showed high class-wise recall for Habit (89.3%), Normal (90.6%), and OSMF (94.0%), indicating balanced discrimination across all categories. Random Forest similarly demonstrated strong performance, particularly for OSMF detection (94.7% recall). These findings suggest that ensemble learning approaches, especially XGBoost, provide superior predictive capability for multiclass classification of Habit, Normal, and OSMF subjects.

**Figure 4:**
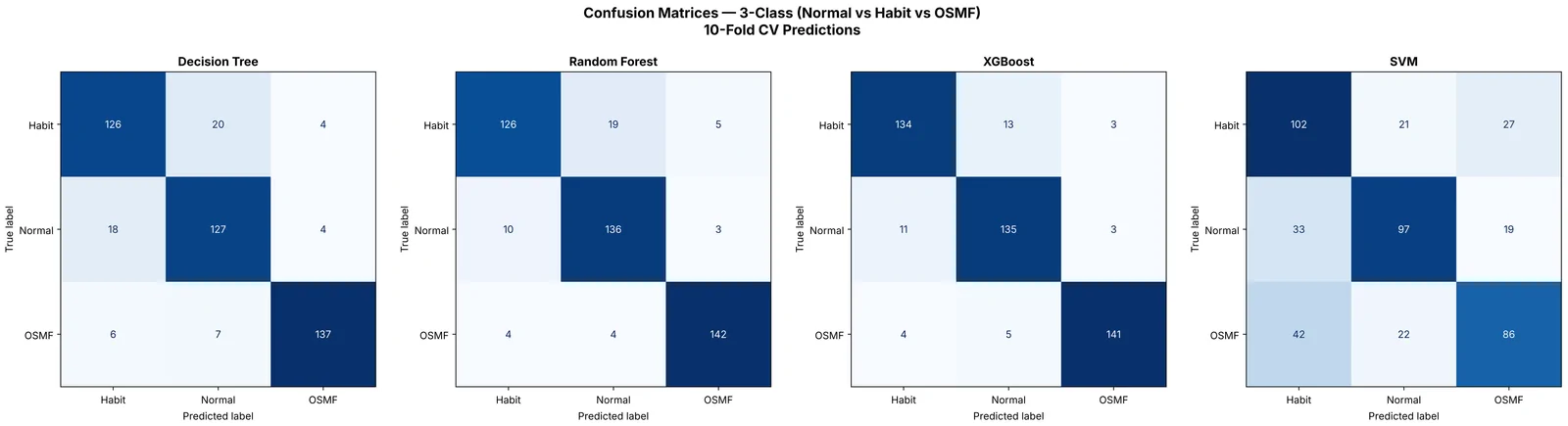
10-fold cross-validation accuracy with standard deviation for all models, three-class comparison.

Ten-fold cross-validation demonstrated superior performance of ensemble-based machine learning algorithms compared with conventional classifiers (**Figure 5**). XGBoost achieved the highest mean accuracy (0.9130 ± 0.0453), F1-score (0.9131 ± 0.0452), recall (0.9130), and specificity (0.9565). Random Forest showed comparable performance with a mean accuracy of 0.8996 ± 0.0420 and specificity of 0.9498. Decision Tree achieved moderate performance (accuracy = 0.8684 ± 0.0501), whereas SVM demonstrated substantially lower classification capability (accuracy = 0.6349 ± 0.0652). Overall, ensemble learning approaches exhibited superior predictive performance and generalizability, with XGBoost emerging as the best-performing classifier (**Supplementary 1**).

**Figure 5:**
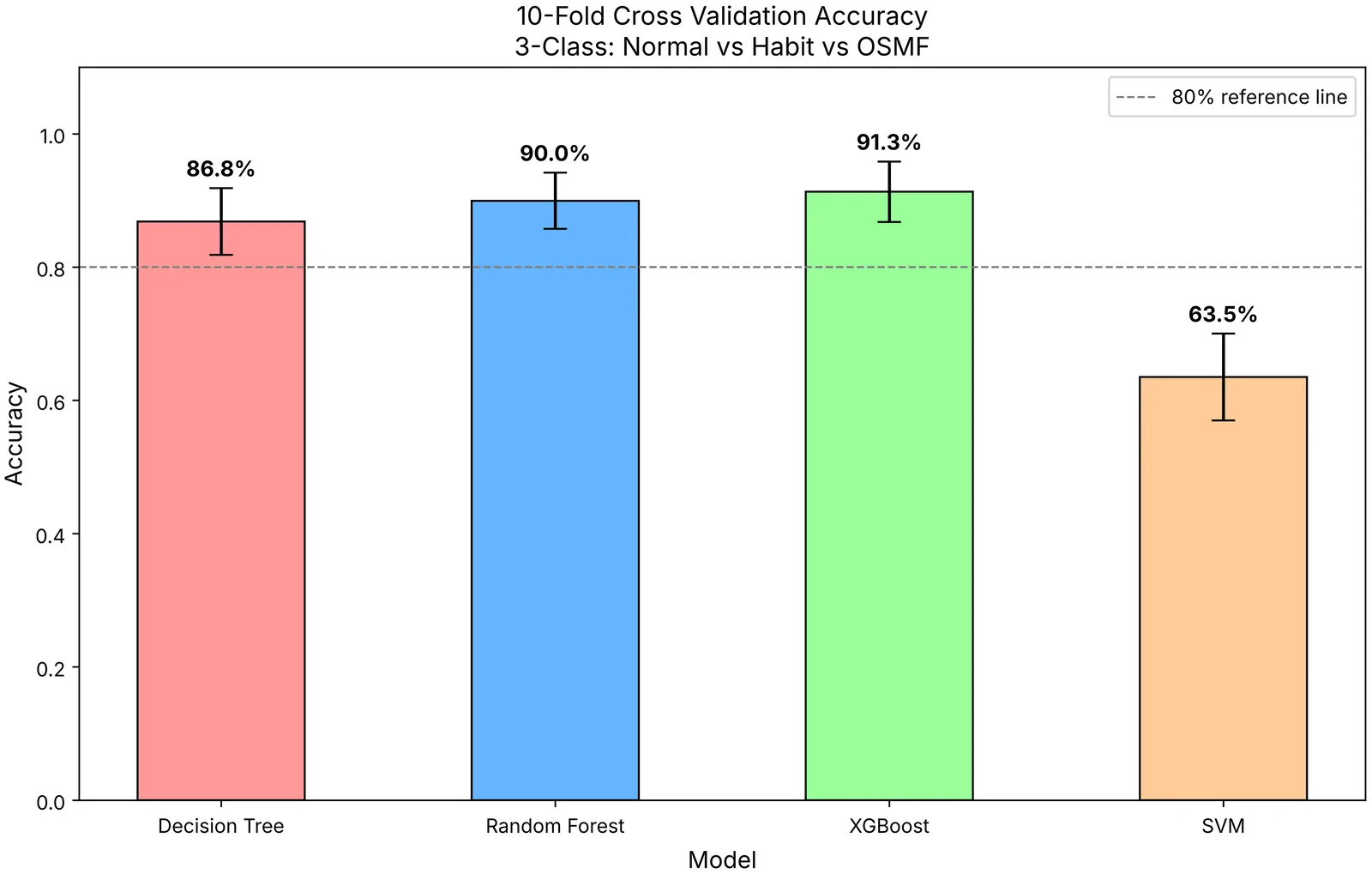
Confusion matrices on the three-class test set.

Bootstrap resampling (1,000 iterations) confirmed the robustness of model performance estimates. XGBoost demonstrated the narrowest confidence intervals for accuracy, F1-score, and recall, indicating stable predictive performance across repeated sampling. The estimated accuracy of XGBoost ranged from 91.5% to 95.8%, outperforming all other evaluated classifiers (**Supplementary 2**).

### 3.5 Performance of the XGBoost model on the internal test dataset

Per-class performance of the XGBoost model is shown in **Table 4**. The classification model demonstrated good overall performance, achieving a weighted average precision, recall, and F1-score of 0.88 on the test dataset (n = 90). Among the individual classes, the Normal category showed the highest recall (0.93), while the OSMF category exhibited the highest precision (0.93). The Habit class achieved a precision and recall of 0.83 each, indicating relatively greater classification difficulty. F1-scores ranged from 0.83 to 0.90 across classes, suggesting consistent and robust predictive performance.

**Table 4:** Per-class performance — XGBoost, three-class.

Class	Precision	Recall	F1-Score	Support
Habit	0.83	0.83	0.83	30
Normal	0.88	0.93	0.90	30
OSMF	0.93	0.87	0.90	30
Weighted Avg	0.88	0.88	0.88	90

### 3.6 ROC curve

One-vs-rest ROC analysis demonstrated excellent discriminatory performance for the ensemble learning models. XGBoost achieved the highest AUC values across all three classes, with ROC curves closely approximating the upper-left corner of the plot. Random Forest showed similarly strong discrimination, whereas Decision Tree exhibited slightly lower but acceptable performance. In contrast, SVM demonstrated substantially reduced discriminatory ability, with ROC curves lying closer to the diagonal reference line. These findings corroborate the cross-validation and confusion matrix analyses, further supporting XGBoost as the optimal classifier for multiclass prediction of Habit, Normal, and OSMF subjects (**Figure 6**).

**Figure 6:**
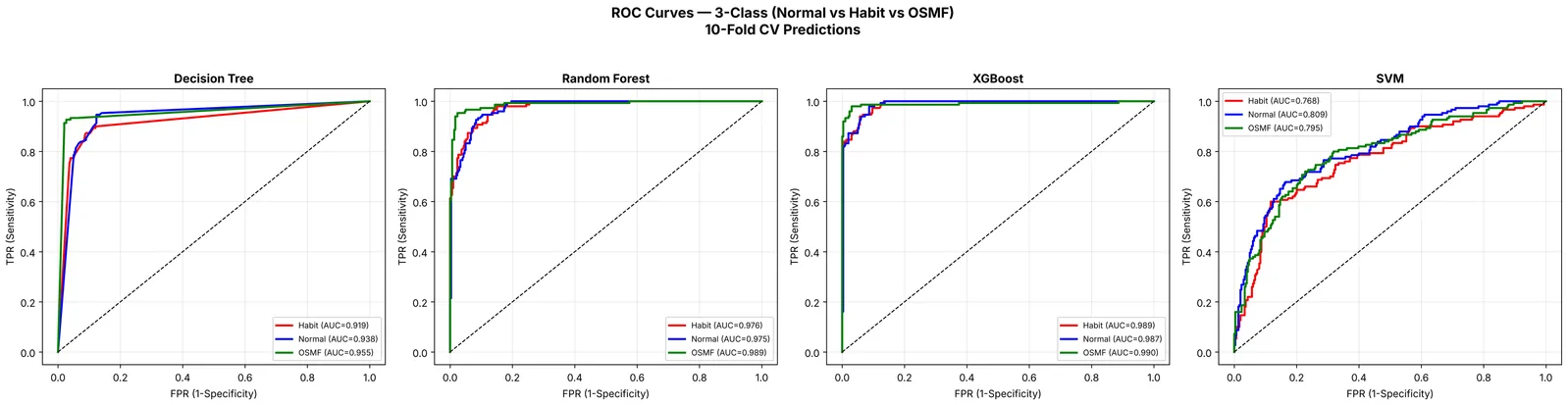
ROC curves for three-class classifiers (10-fold CV).

### 3.7 Normal vs OSMF classification

In the normal versus OSMF classification task, XGBoost achieved the highest mean cross-validation accuracy (97.67% ± 4.22%) and an independent test accuracy of 96.67%. Random Forest demonstrated comparable performance (97.00% ± 3.14%), whereas Decision Tree achieved a cross-validation accuracy of 95.32% ± 4.99%. SVM exhibited substantially lower performance (78.60% ± 7.77%). On the test set, the optimized XGBoost model achieved precision values of 0.94 and 1.00 for normal and OSMF classes, respectively, with corresponding recall values of 1.00 and 0.93. The weighted average precision, recall, and F1-score were each 0.97, indicating excellent classification performance. Bootstrap confidence intervals further confirmed the robustness of the ensemble-based models, while ROC analysis demonstrated near-perfect discrimination between normal controls and OSMF subjects (**Supplementary 3**).

### 3.8 Normal vs habit classification

For discrimination between normal controls and habit users, the optimized XGBoost classifier achieved the highest cross-validation accuracy (90.62% ± 5.20%) and an independent test accuracy of 91.67%. Random Forest demonstrated comparable performance (89.62% ± 5.29%), followed by Decision Tree (85.94% ± 7.45%), while SVM achieved substantially lower accuracy (73.59% ± 4.77%). On the test set, XGBoost achieved precision values of 0.93 and 0.90 for the habit and normal classes, respectively, with corresponding recall values of 0.90 and 0.93. The weighted average precision, recall, and F1-score were all 0.92, indicating balanced classification performance across both groups. Bootstrap confidence intervals confirmed the robustness of the model, while ROC analysis demonstrated strong discriminatory capability for distinguishing habit users from healthy controls (**Supplementary 4**).

### 3.9 External validation of the XGBoost model

The final XGBoost model was further evaluated using an independent external validation cohort comprising 90 participants, with 30 participants in each diagnostic group. For OSMF versus normal control classification, the model achieved an accuracy of 80.0%, sensitivity of 83.9%, specificity of 75.9%, precision of 78.8%, and F1-score of 81.3%. For habit versus normal control classification, performance was lower, with an accuracy of 71.7%, sensitivity of 68.6%, specificity of 76.0%, precision of 80.0%, and F1-score of 73.8%. The detailed performance metrics are presented in **Table 5**. These findings indicate satisfactory generalizability of the developed model, with stronger discriminatory capability for OSMF detection than for distinguishing habit users from healthy controls.

**Table 5:** Performance of the optimized machine learning classifier on an independent external validation cohort.

Metric	OSMF vs normal	Habit vs normal
Accuracy	80.0%	71.7%
Sensitivity	83.9%	68.6%
Specificity	75.9%	76.0%
Precision	78.8%	80.0%
F1-score	81.3%	73.8%
NPV	81.5%	63.3%

## 4 Discussion

The present study evaluated the ability of salivary ATR-FTIR spectroscopy combined with machine learning to differentiate patients with clinically diagnosed OSMF, individuals with adverse oral habits, and healthy controls. The results demonstrate that multivariate analysis of salivary spectral profiles can provide useful classification across these groups. Among the four classifiers evaluated, XGBoost showed the highest performance for the evaluated binary and three-class tasks. The independent validation cohort showed lower performance than the development dataset, particularly for Habit versus Normal classification, emphasizing that the observed classification performance should be interpreted as preliminary evidence of feasibility rather than as established diagnostic accuracy.

The biological basis for differences in salivary spectra between OSMF and healthy individuals is plausible because OSMF is characterized by chronic inflammation, altered extracellular-matrix turnover and progressive fibrosis (10, 11). These processes may alter the abundance or composition of salivary proteins, lipids and other soluble biochemical constituents (12, 13). The statistically identified region from 1581 to 2142 cm^−^¹ therefore provides a potentially relevant multivariate spectral signature. The 1581–1700 cm^−^¹ portion overlaps protein-associated amide vibrations, while other portions may contain carbonyl-related, lipid-associated, and other molecular contributions. The overlap of the nominally significant region with amide-associated vibrations is consistent with the fibrotic nature of OSMF and supports the concept that FTIR can capture disease-related biochemical signatures in saliva. However, the spectral region should not be interpreted as a single molecular biomarker. Although individual wavenumbers did not remain significant after strict Bonferroni correction, the clustering of nominally significant features within a biologically relevant region suggests coordinated biochemical alterations rather than isolated spectral changes. The findings therefore support the presence of a broader spectral pattern rather than establishing a specific disease-associated molecular peak.

The 2000–2142 cm^−^¹ portion requires particular caution. This interval does not correspond to the conventional amide I or amide II regions. Overtone or combination vibrations and contributions from multiple biochemical constituents may contribute to this part of the spectrum, but the present study did not undertake targeted spectral assignment or biochemical confirmation. Consequently, this region should be regarded as a candidate spectral feature requiring further investigation rather than as definitive evidence of collagen or fibrosis-specific absorption. This cautious interpretation is also consistent with the complex composition of saliva, in which proteins, lipids, carbohydrates, nucleic acids, electrolytes and cellular or microbial components may contribute to the measured spectrum.

Principal component analysis provided further evidence that the observed differences were distributed across the multivariate spectral profile. PC1 explained 89.26% of the total variance and PC2 explained 6.95%, together accounting for 96.21% of the variance. The separation in the PCA space appeared to be driven largely by broad differences in overall absorbance and by regions associated with protein and lipid-related spectral features. Importantly, PCA components are mathematical combinations of multiple correlated wavelengths and should therefore not be interpreted as individual biochemical biomarkers.

The machine-learning strategy was designed to compare complementary modeling approaches. Decision Tree provided an interpretable tree-based classifier; Random Forest assessed an ensemble of decision trees and can capture non-linear relationships while reducing the instability of a single tree; SVM with an RBF kernel provided a non-tree-based approach capable of modeling non-linear class boundaries; and XGBoost provided a gradient-boosting approach that can capture interactions and complex patterns across high-dimensional spectral variables. The stronger performance of XGBoost and Random Forest relative to the other models suggests that ensemble tree-based methods were better suited to the multivariate patterns present in this dataset. Ensemble gradient boosting methods are well suited to high-dimensional biomedical data because they can model non-linear relationships, interactions between variables, and subtle patterns distributed across multiple features. In contrast, the lower performance of SVM in this dataset suggests that the spectral differences among groups may not be adequately represented by a simpler decision boundary after PCA reduction. The relatively strong performance of Random Forest and XGBoost indicates that ensemble learning approaches are particularly effective for salivary FTIR classification tasks.

The distinction between normal controls and OSMF was especially robust, with XGBoost achieving near-perfect discrimination in internal evaluation and strong performance on the independent test set. Clinically, this is encouraging because the primary diagnostic challenge is often identifying established disease in a non-invasive manner. The normal-versus-habit classification showed lower performance, reflecting the fact that individuals with adverse oral habits may already exhibit biochemical changes that partially overlap with early disease processes. This overlap is biologically expected and may represent a continuum from exposure-related alterations to overt fibrosis.

An important consideration is the influence of demographic and behavioral factors on the spectral profiles. The groups were age matched, and the OSMF and Habit groups were also matched for duration of tobacco/areca-nut habit. Nevertheless, age, sex, type and intensity of areca-nut/betel-quid or tobacco exposure, smoking, and oral hygiene may influence salivary biochemical composition independently of OSMF. The marked male predominance in the OSMF and Habit groups is an additional limitation. Because the study was not designed or powered to determine sex-specific spectral effects, the present results should not be interpreted as evidence that the identified spectral patterns are independent of sex.

The identified spectral patterns are broadly consistent with previous work demonstrating that vibrational spectroscopy can detect biochemical alterations associated with oral potentially malignant disorders and oral cancer. Previous investigations have reported the ability of FTIR spectroscopy to detect biochemical alterations associated with oral potentially malignant disorders and oral cancer. Banerjee et al. utilized FTIR spectroscopy and SVMs to classify oral leukoplakia and OSCC tissues, achieving a sensitivity of 81.3%, specificity of 95.7%, and an overall accuracy of 89.7% (14). Their use of the Feature Forward Selection method to identify six spectral features (1782, 1713, 1665, 1545, 1409, and 1161 cm^−^¹) was critical for classification. A retrospective analysis of high-risk Oral epithelial dysplasia biopsy samples, employing PCA-LDA for classification was conducted. They achieved a sensitivity of 79 ± 5% and specificity of 76 ± 5%, identifying six key wave numbers for prediction (15). Khan Mohammadi Khorrami et al. analyzed saliva samples for OSCC detection using PLS-DA and SVM-C, achieving accuracies of 0.960 and 0.962, with a sensitivity of 1.00 for OSCC classification (16). Zlotogorski-Hurvitz et al. evaluated FTIR spectra of exosomes from saliva for OSCC detection, reporting a sensitivity of 100%, specificity of 89%, and an overall accuracy of 95% using PCA-LDA. Their focus on salivary exosomes as a diagnostic tool is complementary to our study, which emphasizes whole saliva based FTIR analysis. Both studies reinforce the diagnostic potential of FTIR spectroscopy (17). The study by Rai et al. used FTIR spectroscopy combined with chemometric techniques to differentiate serum metabolic signatures distinguishing OSF patients from controls. FTIR identified 19 key serum peaks (1020–1725 cm^−^¹), which are somewhat similar to the peaks identified in our study. The slight differences may be attributed to variations in the sample type. Chemometric analyses (PCA, HCA, and PLS-DA) confirmed metabolic differences in carbohydrates, proteins, lipids, and nucleic acids, highlighting FTIR’s potential for the rapid diagnosis of OSMF (18).

While their results demonstrate a robust application of other models, our study highlights the advantages of XGBoost in classification of OSMF from normal control and also habit from normal control. The present study extends this body of work by focusing specifically on saliva from OSMF patients and by systematically comparing multiple machine learning classifiers within a standardized pipeline. The use of PCA-based dimensionality reduction, 10-fold stratified cross-validation, and external validation provides a more rigorous analytical framework than many earlier exploratory reports.

A major strength of the study is the inclusion of an independent external validation cohort. While performance decreased compared with internal cross-validation, the model maintained reasonable sensitivity and specificity, particularly for OSMF detection. This drop in performance is common in translational machine learning studies and likely reflects real-world variability in saliva composition, participant characteristics, sample handling, and spectral acquisition. The stronger external performance for OSMF versus normal controls than for habit versus normal controls suggests that established disease produces a more distinct biochemical fingerprint than exposure status alone. Importantly, the external validation results indicate that the model is not simply overfitting the training data. An accuracy of approximately 80% for OSMF detection in an independent cohort suggests meaningful generalizability and supports further multicenter evaluation.

The clinical role of salivary ATR-FTIR should be defined carefully. OSMF in the present study was diagnosed primarily through clinical examination, while histopathological examination was performed when clinically indicated. Histopathology remains important when biopsy is warranted, particularly when dysplasia or malignant transformation is suspected. Accordingly, salivary ATR-FTIR should not be considered a replacement for clinical/oral examination or histopathology. Its potential value lies in providing a non-invasive and repeatable adjunct that could support preliminary screening, risk stratification, or monitoring, particularly where repeated sampling is desirable and access to biopsy, specialist evaluation, or repeated follow-up is limited. There is potential for screening as demonstrated by the ability to distinguish habit users from healthy controls suggesting possible utility in identifying individuals who warrant closer surveillance. Whether such an approach improves clinical decision-making, reduces unnecessary invasive procedures, or facilitates earlier referral cannot be established from the present study and requires prospective clinical evaluation.

### 4.1 Limitations of the study

Several limitations should be acknowledged. First, the study was conducted at a single center, which may limit generalizability across populations with different dietary practices, habit patterns and sample-handling conditions. Second, OSMF diagnosis was primarily clinical, with histopathology performed when indicated; therefore, some degree of diagnostic misclassification cannot be excluded. Third, the cohort was predominantly male, limiting applicability to female populations. Fourth, although the groups were age matched and the OSMF and Habit groups were matched for habit duration, other behavioral and oral-health factors may have influenced salivary composition. Finally, the machine-learning analysis used the same stratified 10-fold cross-validation framework for hyperparameter optimization and performance estimation rather than a fully nested cross-validation design. The subsequent 80/20 test split was generated after tuning, meaning that the test samples had already contributed to the model-selection process. The reported internal and test-set performance should therefore be interpreted as potentially optimistic upper-bound estimates rather than unbiased estimates from a strictly nested design.

### 4.2 Future directions

Future studies should include larger, multicenter and demographically diverse cohorts with balanced sex representation and detailed characterization of areca-nut, tobacco and smoking exposures and oral-health status. Disease severity and longitudinal progression should also be examined to determine whether spectral changes track fibrosis or clinical stage. Future analytical studies should use strictly nested cross-validation or an entirely held-out test cohort, prespecified preprocessing pipelines, and independent external validation. Integration of spectral information with clinical variables and established salivary biomarkers may further improve classification and clinical utility. Most importantly, prospective studies should determine whether salivary ATR-FTIR can provide clinically useful screening or risk-stratification information in real-world populations.

## 5 Conclusion

In conclusion, salivary ATR-FTIR spectroscopy combined with machine learning demonstrated the ability to differentiate clinically diagnosed OSMF, individuals with adverse oral habits, and healthy controls, with XGBoost showing the highest classification performance in the present dataset. The spectral differences are biologically plausible in the context of inflammation, extracellular-matrix turnover and fibrosis, but the identified 1581–2142 cm^−^¹ interval should be regarded as a candidate spectral region rather than a validated disease-specific biomarker, particularly because individual wavenumbers did not remain significant after Bonferroni correction. Performance on the independent validation cohort was lower than that observed during model development, reinforcing the need for further validation. Salivary ATR-FTIR is therefore best positioned as a complementary, non-invasive screening and risk-stratification approach that may support, but should not replace, clinical/oral examination or histopathological assessment when biopsy is indicated. Larger, balanced and multicenter studies using rigorous independent or nested validation are required before this approach can be considered for routine clinical implementation.

## Data Availability

The raw data supporting the conclusions of this article will be made available by the authors, without undue reservation.
