# Supplementary material for: Machine learning-assisted chemometric analysis of salivary ATR-FTIR spectra for the diagnosis of oral submucous fibrosis
Source: Front Oncol. 2026 Oct 19;16:1927981. doi: 10.3389/fonc.2026.1927981 (PMC13724524; doi:10.3389/fonc.2026.1927981)
Supplement: Supplementary file 1 [file DataSheet1.docx]

| **Model** | **CV Acc Mean** | **CV Acc SD** | **CV F1 Mean** | **CV F1 SD** | **CV Recall Mean** | **CV Specificity Mean** | **CV Time (s)** |
| --- | --- | --- | --- | --- | --- | --- | --- |
| Decision Tree | 0.8684 | 0.0501 | 0.8687 | 0.0498 | 0.8684 | 0.9342 | 6.14s |
| Random Forest | 0.8996 | 0.0420 | 0.8991 | 0.0422 | 0.8996 | 0.9498 | 31.05s |
| SVM | 0.6349 | 0.0652 | 0.6306 | 0.0688 | 0.6349 | 0.8175 | 2.28s |
| XGBoost ★ | 0.9130 | 0.0453 | 0.9131 | 0.0452 | 0.9130 | 0.9565 | 5.75s |

*Supplementary 1. Detailed 10-Fold CV Metrics — Three-Class. ★ = best model.*

| **Model** | **Accuracy 95% CI** | **F1 95% CI** | **Recall 95% CI** |
| --- | --- | --- | --- |
| Decision Tree | [0.864–0.944] | [0.864–0.944] | [0.864–0.944] |
| Random Forest | [0.906–0.953] | [0.905–0.953] | [0.906–0.953] |
| SVM | [0.617–0.693] | [0.615–0.693] | [0.617–0.693] |
| XGBoost ★ | [0.910–0.958] | [0.915–0.958] | [0.915–0.958] |

*Supplementary 2. Bootstrap 95% CIs — Three-Class (n=1,000). ★ = best model.*

| **Model** | **CV Accuracy** | **CV Std** | **Test Accuracy** |
| --- | --- | --- | --- |
| Decision Tree | 87.30% | ±3.24% | — |
| Random Forest | 88.31% | ±5.80% | — |
| SVM | 68.57% | ±12.28% | — |
| XGBoost (tuned) ★ | 97.67% | ±4.22% | 96.67% |

*Supplementary 3a. Normal vs OSMF results (PCA n=10). ★ = best model.*

*Supplementary 3b. 10-fold cross-validation accuracy with standard deviation for all models, Normal vs OSMF comparison.*

| **Class** | **Precision** | **Recall** | **F1-Score** | **Support** |
| --- | --- | --- | --- | --- |
| Normal | 0.94 | 1.00 | 0.97 | 30 |
| OSMF | 1.00 | 0.93 | 0.97 | 30 |
| Weighted Avg | 0.97 | 0.97 | 0.97 | 60 |

*Supplementary 3c. Per-class performance — XGBoost, Normal vs OSMF.*

*Supplementary 3d. Confusion matrices on the Normal vs OSMF test set.*

Supplementary 3e. *ROC curves on Normal vs OSMF (10-fold CV).*

| **Model** | **CV Accuracy** | **CV Std** | **Test Accuracy** |
| --- | --- | --- | --- |
| Decision Tree | 85.95% | ±6.80% | — |
| Random Forest | 86.95% | ±5.26% | — |
| SVM | 72.92% | ±4.27% | — |
| XGBoost (tuned) ★ | 90.62% | ±5.20% | 91.67% |

*S4a. Normal vs Habit results (PCA n=10). ★ = best model.*

*S4b. 10-fold cross-validation accuracy with standard deviation for all models, Normal vs Habit comparison.*

| **Class** | **Precision** | **Recall** | **F1-Score** | **Support** |
| --- | --- | --- | --- | --- |
| Habit | 0.93 | 0.90 | 0.92 | 30 |
| Normal | 0.90 | 0.93 | 0.92 | 30 |
| Weighted Avg | 0.92 | 0.92 | 0.92 | 60 |

*S4c. Per-class performance — XGBoost, Normal vs Habit.*

*S4d. Confusion matrices on the Normal vs Habit test set.*

*S4e. ROC curves on Normal vs Habit (10-fold CV).*
